# Comment on ‘MicroRNA-199b-5p attenuates TGF-β1-induced epithelial–mesenchymal transition in hepatocellular carcinoma’

**DOI:** 10.1038/s41416-018-0013-1

**Published:** 2018-03-19

**Authors:** Ion Cristóbal, Andrea Santos, Silvia González, Melania Luque, Blanca Torrejón, Federico Rojo, Jesús García-Foncillas

**Affiliations:** 1 Translational Oncology Division, Oncohealth Institute, IIS-Fundacion Jimenez Diaz-UAM, University Hospital “Fundacion Jimenez Diaz”, Madrid, Spain; 2 Pathology Department, IIS-Fundacion Jimenez Diaz-UAM, University Hospital “Fundacion Jimenez Diaz”, Madrid, Spain

**Keywords:** Oncogenes, Oncogenes

We read with great interest the recent publication by Zhou and colleagues, which provides novel relevant data about the role of microRNA-199b-5p (miR-199b) and N-cadherin as regulators of epithelial–mesenchymal transition (EMT) in human hepatocellular carcinoma (HCC). In this work, the authors demonstrate that N-cadherin is a direct miR-199b target, and observed that miR-199b downregulation in HCC patients predicts poor outcome and inversely correlates with N-cadherin overexpression. At the functional level, they demonstrated that miR-199b inhibits both migratory and invasive abilities of HCC cells, and metastatic progression in vivo. In addition, they identified a positive regulatory loop between N-cadherin, TGF-β1, and Akt, and showed that miR-199b reduces TGF-β1-induced Akt phosphorylation, thereby affecting EMT in HCC. Despite that this is a very interesting piece of work that provides novel important findings, some considerations should be taken into account.

From our point of view, it would be very important to evaluate the potential relevance of alternative miR-199b targets and even more strongly in the work by Zhou et al. due to the following rationale. The authors showed in their work that the functional effects of miR-199b were only partially reversed by N-cadherin restoration, which highlights the involvement of additional molecular targets. Of note, miR-199b has been reported to target SET,^1,2^ which has been recently confirmed by our group in colorectal cancer, showing that SET is of key relevance for miR-199b-induced effects.^3^

Interestingly, SET has been reported to have a relevant role in EMT in pancreatic cancer via N-cadherin regulation. SET overexpression was found to activate signaling through Rac1/JNK/c-Jun, thereby inducing the transcriptional activation of N-cadherin expression. In fact, SET overexpression positively correlated with N-Cadherin levels.^4^ These observations would indicate that the direct miR-199b-induced N-cadherin regulation would be indirectly reinforced by modulating SET.

Furthermore, the authors showed that miR-199b predicted poor outcome in their HCC cohort and reduced TGF-β1-induced Akt phosphorylation. In concordance, SET overexpression has been described to confer poor clinical outcome and correlate with p-Akt expression in HCC tumors.^5^ SET is a potent endogenous inhibitor of the tumor suppressor protein phosphatase 2A (PP2A), and PP2A activation has been reported to suppress TGF-β1-induced EMT.^6^ In addition, PP2A directly targets and dephosphorylates Akt, which has also been described in HCC.^7^ In fact, SET targeting using an antagonist to this protein restored PP2A-mediated p-Akt downregulation and induced apoptosis of HCC cells.^5^

Finally, Zhou et al. found that miR-199b inhibits both migration and invasion in HCC cells, and some evidence highlights the potential involvement of SET in these effects. First, SET has a role inducing Rac1-mediated migration.^8^ Second, a recent work has described that matrix metalloproteinase-9 (MMP-9) deregulation through PP2A inhibition is a relevant event in HCC that contributes to the tumor invasion,^9^ and the PP2A inhibitor SET has been reported to induce MMP-9 expression activity in breast and non-small cell lung cancer.^10,11^ Altogether, the work by Zhou et al.^12^ suggest that miR-199b/N-cadherin deregulation is a relevant event that predicts poor outcome and contributes to EMT in HCC. However, forthcoming studies are warranted to confirm the wide range of evidence indicating that SET could also be having a key role in the miR-199b/N-cadherin interplay.
